# Current status and perspectives on the use of androgen receptor pathway inhibitors in the salvage radiotherapy setting

**DOI:** 10.1038/s41391-024-00878-0

**Published:** 2024-08-13

**Authors:** Vérane Achard, Thomas Zilli

**Affiliations:** 1 Radiation Oncology, HFR Fribourg, Villars-sur-Glâne, Switzerland; 2 Faculty of Medicine, University of Geneva, Geneva, Switzerland; 3 Radiation Oncology, Oncology Institute of Southern Switzerland (IOSI), EOC, Bellinzona, Switzerland; 4 Faculty of Biomedical Sciences, Università della Svizzera Italiana, Lugano, Switzerland

**Keywords:** Cancer therapy, Cancer therapy

Salvage radiotherapy (sRT) after radical prostatectomy (RP) is the standard of care treatment for prostate cancer (PCa) patients experiencing biochemical recurrence (BCR). Delivery of early sRT (initiated at prostate-specific antigen (PSA) level below 0.25 ng/mL) is associated with a reduction in all-cause mortality risk compared to men who received sRT when PSA levels are above this threshold [1]. Treatment intensification is needed for this latter group of patients and those with other unfavorable disease characteristics (such as a rapid PSA doubling time - PSA-DT and ISUP group ≥ 4) to improve clinical outcomes.

In contrast to the primary radiotherapy setting, the benefit of hormone therapy in the sRT setting is less well-defined. Four randomized controlled trials (RCTs) investigated the benefit of adding hormone therapy to sRT (GETUG-AFU 16, RTOG 9601, SPPORT/RTOG 0534, RADICALS-HD). The GETUG-AFU 16 and SPPORT trials showed that the addition of a short-term androgen deprivation therapy (ADT) to sRT delays PSA progression-free survival (PFS). A post-hoc analysis of the GETUG-AFU 16 also demonstrated a 6% improvement in metastasis-free survival (MFS) at 5 and 10 years but no overall survival (OS) benefit [2]. The RTOG 9601 was the only RCT demonstrating an OS benefit from adding hormone therapy [3]. Interestingly, it didn’t use ADT but bicalutamide 150 mg, a first-generation androgen receptor pathway inhibitor (ARPI) for two years. Twelve years OS was 76.3% in the bicalutamide group and 71.3% in the placebo group (HR 0.77; 95% CI 0.59–0.99; *p* = 0.04). Finally, RADICALS-HD randomized 2839 patients with BCR to postoperative RT with none, 6, or 24 months of ADT [4, 5**]**. The authors reported that adding 6 months of ADT to postoperative RT did not improve MFS compared with no ADT while adding 24 months of ADT improved MFS when compared to 6 months of ADT. The benefit remained marginal, with the 10-year MFS being 71.9% in the 6-month ADT group and 78.1% in the 24-month ADT group. Overall, the DADSPORT meta-analysis on these 4 trials showed no evidence of an OS benefit with hormone therapy vs. none, irrespective of whether 6 months or 24 months of hormonal suppression [6].

ARPI have profoundly impacted the management of metastatic PCa. Recent studies indicate that they will likely impact the treatment of high-risk localized PCa. Abiraterone increased OS of very high-risk or node-positive PCa treated by RT and ADT [7, 8]. In the EMBARK trial, enzalutamide, in monotherapy or with ADT, increased MFS of high-risk BCR patients, i.e. with a PSA-DT ≤ 9 months and a PSA ≥ 2 ng/mL above nadir after RT or ≥ 1 ng/mL after RP with or without postoperative RT [9]. Therefore, it is not surprising that the ARPI enthusiasm has reached the sRT setting in an attempt to further improve the clinical outcomes of patients experiencing BCR. Results of five prospective phase II trials have been recently presented, as summarized in Table 1. The comparison of these trials is hampered by the lack of a harmonized definition of biochemical relapse at inclusion and the absence of a standardized primary endpoint, although all studies used a PSA-based primary endpoint. Two single-arm trials evaluated the addition of either abiraterone or enzalutamide for 6 months to sRT and ADT [10, 11], while three trials randomized patients to receive sRT with intensified systemic therapy using enzalutamide alone vs. observation (SALV-ENZA) [12], abiraterone, apalutamide, and ADT for 6 months vs. complete androgen blockade for 6 months (FORMULA-509) [13], and enzalutamide and ADT for 24 months vs. ADT for 24 months (RTOG 3506-STEEL) [14]. While the single-arm studies included patients with BCR without further refinement of patient selection criteria, in the three RCTs, only patients with high-risk BCR were randomized, though the definition of high-risk relapse differed between the two studies. Notably, in the CARLHA trial, the 3-year biochemical relapse-free survival was 81.5% [10], whereas in the STREAM trial, the 2-year PFS was 65% [11]. The worse pathology characteristics in the STREAM trial compared to the CARLHA trial may explain these worse results (21.1% pN1 and 47.3% ISUP group ≥ 4 in the STREAM trial vs. 0% pN1 and 15% ISUP group ≥ 4 in the CARLHA trial). Compared to standard treatment, in the three RCTs, the addition of ARPI improved PSA progression, as expected by the deeper and longer testosterone suppression. Interestingly, in a pre-planned analysis by the stratification factors of the FORMULA-509 trial, there was a significant benefit in 3-year MFS (66.1% vs. 84.3%, HR 0.32 (90% CI 0.13–0.84), *p* = 0.02) for patients with PSA > 0.5 ng/mL. This result is in agreement with RTOG 9601 findings, where only patients with a PSA > 0.6 ng/mL derived an OS benefit of the addition of long-term bicalutamide [3]. Many other trials investigating the addition of an ARPI to sRT are ongoing, including several phase 3 trials, some of which are shown in Table 2.

**Table 1:** Available prospective studies on the use of ARPI in the salvage RT setting.

First Author Year Trial name	Phase	Definition of BCR and patient eligibility	No pts	Pts characteristics	Median FU (months)	Arm 1	Arm 2	Primary outcome	Grade ≥ 3 toxicity CTCAE v4.0
Posadas 2024 STEEL *Abstract* [14]	2 Randomized	PSA ≥ 0.2 ng/mL post-RP and ≥ 1 unfavorable feature: - Gleason score 8-10 - seminal vesicle invasion - pN1 - persistent PSA > 0.1 ng/mL after RP - PSA ≥ 0.7 ng/mL	188	- Median PSA = NA - Median PSADT = NA - ISUP group > 4 = 52% - R1 = NA - pN1 = 22%	15.8	RT (66.0–70.2 Gy) + 24 mo ADT	RT (66.0–70.2 Gy) + 24 mo ADT/ enzalutamide	**PFS** Time from randomization to the date of progression, death from any cause or last known follow-up date HR = 0.72, 80% CI:0.56-0.94, *p* = 0.14, in favor of Arm 2	15% Arm 1 24% Arm 2
Ah-Thiane 2024 CARLHA [10]	2 Single-arm	- PSA ≤ 0.1 ng/mL within 6 mo after RP - PSA between 0.2 and 2 ng/mL at inclusion - pN1 excluded	46	- Median PSA = 0.36 ng/mL - Median PSADT = 7.5 mo - ISUP group ≥ 4 = 15% - R1 = 50% - pN1 = 0%	47	RT (66 Gy/33 fr) + 6 mo ADT / abiraterone	None	**3y-BCRFS** Time from inclusion to PSA ≥ 0.2 ng/mL or death from any cause 81.5%	Radiotherapy-related: 11% Non-Radiotherapy-related: 43%
Nguyen 2023 FORMULA-509 *Abstract* [13]	2 Randomized	PSA ≥ 0.1 ng/mL post-RP and ≥ 1 unfavorable feature: - Gleason score 8-10, - PSA > 0.5 ng/mL - pT3/T4 - pN1 or radiographic N1 - PSADT < 10 mo - Negative margins - Persistent PSA - Gross local/regional disease - Decipher High Risk	345	- Median PSA = 0.3 ng/mL - Median PSADT = NA - ISUP group ≥ 4 = 46.1% - R1 = NA - pN1 = 28.7%	34	RT + 6 mo ADT / bicalutamide	RT + 6 mo ADT / abiraterone / apalutamide	**3y-PSA PFS** Time from randomization to PSA failure or death from any cause 68.5% (arm 1) *vs.* 74.9% (arm 2)	NA
Tran 2022 SALV-ENZA *Abstract* [12]	2 Randomized	- One rise in PSA above a baseline detectable value (≥0.05 ng/mL) using measurements taken at least 4 weeks apart from each other - PSA between 0.1 and 0.7 ng/mL at inclusion - pathological Gleason sum 8–10; or pathological Gleason sum 7 and either pT3 or R1 disease	86	- Median PSA = 0.3 ng/mL - Median PSADT = NA - ISUP group ≥ 4 = 45% - R1 = 50% - pN1 = 0%	34	RT (66.6–70.2 Gy /37–39 fr) + placebo	RT (66.6–70.2 Gy/37–39fr) + 6 mo enzalutamide monotherapy	**2y-FFPP** Time from randomization to the date of PSA progression 66% (Arm 1) *vs*. 84% (Arm 2)	NA
Bitting 2021 STREAM [11]	2 Single-arm	PSA > 0.2 ng/mL, with 2 consecutive rises over the post-RP nadir, or failure to obtain the PSA nadir of <0.2 ng/mL after RP	38	- Median PSA = 0.4 ng/mL - Median PSADT = NA - ISUP group ≥ 4 = 47.3% - R1 = 47.3% - pN1 = 21.1%	37.5	RT (66 Gy/33 fr) + 6 mo ADT / enzalutamide	None	**2y-PFS** PFS was defined as the proportion of individuals with testosterone >100 ng/dl at 24 mo after registration without (1) PSA ≥ 0.2 ng/mL above the post-RT nadir (2) continued consecutive rise in the PSA level following treatment if no nadir was experienced (3) evidence of clinical progression or initiation of therapy for progressive disease (4) death 65%	23%

*ADT* androgen deprivation therapy, *BCR* biochemical relapse, *BCRFS* biochemical relapse free survival, *CI* confidence interval, *FFPP* Freedom-from-PSA-Progression, *fr* fraction, *Gy* Gray, *MFS* metastases-free survival, *mo* month, *NA* non applicable, *PFS* progression-free survival, *pts* patients, *PSADT* PSA doubling time, *RP* radical prostatectomy, *y* year

**Table 2:** Trials on the use of ARPI in the salvage RT setting.

Trial name NCT number Trial status	Phase	Definition of BCR and patient eligibility	No pts	Arm 1	Arm 2	Primary outcome
**NRG GU008** NCT04134260 Recruiting	3	• Detectable PSA after RP. Detectable PSA is defined as serum PSA > 0 ng/mL at least 30 days after prostatectomy • pN+ patients	586	RT + 24 mo ADT	RT + 24 mo ADT/apalutamide	MFS
**DASL-HiCaP** NCT04136353 Active, not recruiting	3	High-risk localized PCa or High-risk BCR • PSA ≥ 0.1 ng/mL that has risen or remained stable • Grade Group 5, OR • Grade Group 4 AND pT3a or higher, OR • Pelvic nodal involvement defined radiologically as greater than 10 mm on short axis using standard CT or MRI, or pathologically confirmed (PSMA PET alone is not considered enough if ≤ 10 mm)	1100	RT + 8 mo ADT/placebo	RT + 8 mo ADT/ darolutamide	MFS
**SAVE** NCT03899077 Recruiting	2 Randomized	PSA detectable with confirmed rise (at least 2 weeks apart) at least 8 weeks after RP.	202	RT + 6 mo ADT	RT + 6 mo apalutamide	EPIC-26 sexual domain score 9 mo after start of hormonal treatment
**BALANCE (NRG-GU006)** NCT03371719 Active, not recruiting	2 Randomized	• Post-RP patients with a detectable serum PSA ( ≥0.1, but ≤1.0 ng/mL) at study entry (within 90 days of Step 1 registration) and at least one of the following: ∘ Gleason score 7–10 (International Society of Urological Pathology [ISUP] grade group 2 to 5) ∘ ISUP grade group: ▪ Grade Group 1: Gleason score ≤ 6, ▪ Grade Group 2: Gleason score 3 + 4 = 7, ▪ Grade Group 3: Gleason score 4 + 3 = 7, ▪ Grade Group 4: Gleason score 8, ▪ Grade Group 5: Gleason scores 9 and 10 ∘ ≥T3a disease ∘ Persistent elevation of PSA after prostatectomy measured within 90 days after RP (PSA never became undetectable) of > 0.04 but < 0.2 ng/mL (PSA nadir) • pN0 or pNx	311	RT + 6 mo placebo	RT + 6 mo apalutamide	Biochemical PFS
**PRIMORDIUM** NCT04557059 Recruiting	3	• High-risk BCR patients: ∘ any post-operative prostate-specific antigen (PSA) measurement < 0.1 ng/mL within 12 mo after RP and without any PSA ≥ 0.1 ng/mL within the 4 to 8-week period after RP ∘ Gleason score ≥ 8 OR ∘ PSADT ≤ 12 mo • Persistent PSA after RP: ∘ PSA ≥ 0.1 ng/mL within the 4 to 8-week period after RP, confirmed by additional measurement at least 3 weeks later ∘ Gleason score ≥ 8	412	RT + 6 mo ADT	RT + 6 mo ADT/apalutamide	Prostate specific Membrane Antigen-Positron Emission Tomography (PSMA-PET) Metastatic Progression-free Survival
**CARLHA-2** NCT04181203 Recruiting	3	• PSA ≥ 0.2 ng/mL at the time of randomization with an elevation of PSA over three consecutive assays • PSA at relapse > 0.5 ng/mL or • Gleason score >7 or tumor stage pT3b or • resection margins R0 or • PSADT ≤ 6 mo or pelvic lymph node relapse (N1, ≤ 5 lymph nodes)	490	RT + 6 mo ADT	RT + 6 mo ADT/Apalutamide	PFS

*ADT* androgen deprivation therapy, *BCR* biochemical relapse, *MFS* metastases-free survival, *mo* months, *PFS* biochemical progression-free survival, *pts* patients, *RT* radiotherapy, *PCa* prostate cancer, *PSA* prostate specific antigen, *PSADT* PSA doubling time.

Although the treatment association with ARPI remains potentially appealing, clear identification of the patients who can really benefit from treatment intensification remains primordial. In a post-hoc subgroup analysis of the RTOG 9601 trial, the most remarkable OS benefit was observed in men with an elevated PSA level at entry above 1.5 ng/mL. In contrast, the median pre-sRT PSA was much lower in the GETUG-AFU 16 (0.3 ng/mL), SPPORT (0.35 ng/mL), and RADICALS-HD (0.2 ng/mL), all studies unable to demonstrate an OS benefit by adding 4-6 months of ADT. Pre-sRT PSA is currently the most important prognostic risk factor at the time of recurrence and sRT. Other prognostic risk factors, such as PSA-DT and pathological ISUP grade have been identified in a systematic review [15] and form the basis for the EAU BCR risk group stratification. It is likely that additional factors, such as the timing of recurrence and, hopefully, soon, genomic classifiers and artificial intelligence-based predictive models on digital pathology, will further refine this stratification and guide treatments.

In conclusion, patient selection for concomitant and adjuvant systemic therapies in the sRT setting is crucial. Future trials investigating the use of ARPI in combination with sRT should focus exclusively on very high-risk BCR patients to avoid incurring high toxicity for a marginal benefit. As such, an unknown proportion of patients in the five trials may have been overtreated [10–14]. Moreover, use of stronger and more robust outcome endpoints compared to PSA-based measures should be encouraged when exploring the role of ARPI in the salvage setting. Overall, we believe that the use of ARPI with or without ADT may represent a tremendous opportunity to improve the therapeutic ratio in these high-risk BCR patients who are candidates for sRT not only to potentially improve MFS, as shown in the EMBARK study [9], but also to allow de-escalation in men who are candidates for 24 months of hormonal manipulation. Last but not least, use of ARPI in monotherapy, as explored in the SALV-ENZA study [12], could offer the possibility of better sparing sexual function and bone mineral density compared with standard ADT, reducing quality of life impact of systemic therapies [16], particularly in young men who wish to preserve sexual function.
